# Hygrothermal Durability and Damage Evolution of Bio-Epoxy-Based Composites Reinforced with Different Fibre Types

**DOI:** 10.3390/polym18010058

**Published:** 2025-12-25

**Authors:** Abdullah Iftikhar, Allan Manalo, Zaneta Senselova, Wahid Ferdous, Mazhar Peerzada, Hannah Seligmann, Kate Nguyen, Brahim Benmokrane

**Affiliations:** 1 Centre for Future Materials, University of Southern Queensland, Toowoomba, QLD 4350, Australia; abdullah.iftikhar@unisq.edu.au (A.I.); zaneta.senselova@unisq.edu.au (Z.S.); wahid.ferdous@unisq.edu.au (W.F.); mazhar.peerzada@unisq.edu.au (M.P.); hannah.seligmann@unisq.edu.au (H.S.); 2 School of Engineering, RMIT University, GPO Box 2476, Melbourne, VIC 3001, Australia; kate.nguyen@rmit.edu.au; 3 Department of Civil and Building Engineering, University of Sherbrooke, Sherbrooke, QC J1K 2R1, Canada; brahim.benmokrane@usherbrooke.ca

**Keywords:** hygrothermal durability, material degradation, bio-composites, bio-based polymers, environmental ageing, fracture behaviour

## Abstract

This study investigates hygrothermal durability of bio-epoxy composites reinforced with carbon, E-glass, basalt, and flax fibres. Fibre yarns and bio-composites were exposed for 3000 h at 60 °C and 98% relative humidity. The tensile strength reduction in the fibres and the interfacial shear strength (IFSS) reduction in the composites were assessed after ageing. Chemical deterioration was evaluated using energy-dispersive X-ray spectroscopy (EDS); morphological changes in fibres and composites fracture surfaces were examined using a scanning electron microscope (SEM). Results indicated that the durability was significantly influenced by fibre types. Tensile strength reduction was higher in carbon, glass and basalt compared to flax yarns because of chemical degradation of the sizing layer in synthetic fibres, while only physical damage was observed in flax. The IFSS reduction was highest in flax composites (10%), and lowest in carbon (4%). EDS indicated the hydrolysis and erosion of fibre sizing, with reduced silica content in glass and basalt fibres. SEM revealed matrix-dominated failure in carbon/bio-epoxy, interfacial debonding in glass and basalt composites, fibre slip and pull-out in flax/bio-epoxy. Overall, the results highlighted damage propagation pathways and demonstrated that bio-epoxy composites exhibited reasonable performance under hygrothermal ageing, supporting their potential as a sustainable alternative in durability-critical applications.

## 1. Introduction

Interest in the use of fibre-reinforced polymer (FRP) composites has increased because of their corrosion resistance, light weight, high strength-to-weight ratio, and high stiffness-to-weight ratio [1,2]. In civil engineering applications, FRP composites are now commonly used in bridge decks, railway sleepers, building facades, marine structures, wind turbine blades, and electrical transmission lines [3,4,5,6,7]. Various synthetic and natural fibres are used to manufacture FRP composites, depending on the intended use during service life. Synthetic fibres are preferred for highly demanding structural applications. For instance, glass fibres are used for all composite civil infrastructure and carbon fibres are preferred when the strength requirements are even higher [8]. Basalt fibres have also emerged as a competitive substitute for glass fibres, offering comparable strength and cost while providing immunity to high temperatures [9]. However, the main disadvantages of synthetic fibres compared to natural fibres are their higher costs and greater environmental impact. Moreover, natural fibres are gaining significant consideration as a cost-effective alternative to synthetic fibres owing to their lower density, higher specific strength, higher specific modulus, environmental friendliness, and sustainability [10,11,12]. Among natural fibres, flax fibres are a predominant choice due to their availability and superior mechanical properties compared to other natural fibres [13]. Owing to their excellent chemical, thermal, and mechanical performance, vinyl ester and epoxy resins are widely utilized among thermoset resin systems [14]. The disadvantages related to the thermoset resins include environmental concerns, such as non-recyclability, which increases waste in landfills, and the depletion of crude oil and petroleum resources. Consequently, a growing interest has emerged in bio-based epoxy resins due to their non-toxicity, biodegradability, environmental sustainability, and cost efficiency [14,15]. Replacing petroleum-based resins with a bio-epoxy system supports circular-economy goals by lowering carbon footprint and improving end-of-life sustainability. Throughout their life span, FRP composites are expected to be subjected to severe environmental conditions such as moisture, high temperature [16], or a synergistic effect of moisture and temperature [11,17,18]. Therefore, a comprehensive understanding of how these environmental conditions influence the durability of bio-based polymer composites is crucial for their broader applicability.

Exposure to moisture can lead to changes in the morphology of FRP composites through multiple mechanisms. Matrix swelling generates microcracks, which can facilitate diffusion paths for moisture. Eventually, the moisture can reach the interface of the fibre and matrix, damage the chemical bonding and thereby compromise the stress transfer capabilities [19,20]. Likewise, FRP composites show a poor performance at elevated temperatures [21]. Although the fibres are generally more resistant to the elevated temperature, the resin part softens at temperatures beyond the glass transition temperature. As a result, resin loses stiffness and the ability to hold the fibres, eventually reducing the mechanical performance of composites by reducing the load transfer capabilities between fibre and matrix, as presented in previous studies [16,22,23,24]. It has also been reported that when moisture is coupled with temperature, the moisture absorption is accelerated, which further accelerates the ageing process and degradation of polymer composites [10,13,20]. Elarbi and Wu [25] stated that the extent of damage in polymer composites was greater when subjected to a hygrothermal environment compared to only moisture. Basalt/epoxy composites gained 1.5% weight after immersion in seawater at 40 °C, while glass/epoxy composites exhibited 1.2% moisture uptake under similar conditions [26]. It was also reported that the flexural and interlaminar shear strength (ILSS) of basalt epoxy and glass epoxy composites were adversely affected under hygrothermal ageing. Basalt/epoxy exhibited a decrement of 22% in ILSS compared to glass/epoxy, with a reduction of 20%. Flexural strength reduction of 6% in glass/epoxy and 25% in basalt/epoxy was observed [26]. In another study [27], a weight gain of 0.8% was reported in basalt/epoxy composites after 45 days of water immersion at 40 °C. Basalt/epoxy and E-glass/epoxy composites fabricated following identical manufacturing parameters exhibited approximately 3.5% and 6% increase in weight, respectively, after water immersion for 100 days at 80 °C [28]. Their ILSS was also reported to be reduced by 40% and 60% because of weakened fibre/matrix interface [28]. Wang et al. [29] reported a 3% weight gain in basalt/epoxy composites exposed to moisture for 84 days at 40 °C compared to glass/epoxy, which gained 0.3% weight under identical manufacturing and ageing setup. Das et al. [13] concluded that the moisture absorption behaviour depends on the fibre type, as they observed different water absorption in flax and glass fibres with the same resin subjected to hygrothermal ageing. Scida et al. [30] reported that flax fibre-based epoxy composites exhibited a loss of 12% and 55% in strength and modulus, respectively, after hygrothermal conditioning. The fibre/resin interface is the most critical part in polymer composites because all the stresses are transferred between fibre and resin through this transition region. Many studies revealed that hygrothermal ageing negatively affected the fibre/matrix interface, resulting in reduced overall performance of composites [31,32,33]. Fibre sizing is a crucial component of the interfacial region, and the degradation of the sizing layer negatively influences the stress transfer through the interface [34]. Nikforooz et al. [31] observed that the fibre sizing notably influenced the IFSS by negatively impacting the interface, after using the single fibre fragmentation approach. Zhong et al. [32] performed a study for the comparative evaluation of the performance of glass and carbon FRP under hygrothermal ageing and reported a significant reduction of 26% and 5% in strength, respectively. The higher strength reduction in glass FRP was attributed to the considerable fibre strength reduction (29%) due to the sizing degradation [32].

Previous research shows that hygrothermal ageing negatively impacts FRP composites, with the type of fibre, manufacturing methods, and ageing environments as the major factors influencing the level of degradation. However, most of the available studies involving the evaluation of the durability of FRP composites lack a systematic comparative evaluation of multiple fibre types. Despite the growing interest in the usage of bio-based polymer composites, their durability when combined with various fibre types under the same manufacturing and exposure conditions remains largely undocumented. Although the literature highlights the importance of fibre sizing in the fibre/matrix interface, evaluation of the degradation of the sizing layer and its impact on the reduction in interfacial shear strength and interface damage is scarce. Therefore, a systematic evaluation is needed that can highlight the effects of various fibre types on the long-term durability of bio-epoxy composites under similar fabrication methods and hygrothermal ageing, and to identify the underlying degradation mechanisms, particularly sizing damage and its consequences for the fibre/matrix interface.

The current study includes the assessment of the durability performance of bio-epoxy resin, fibre yarns (i.e., carbon, glass, basalt, flax), and single yarn bio-composites subjected to the simultaneous effect of raised temperature and moisture by implementing an extensive accelerated ageing program. The research is targeted to understand the combined effect of elevated temperature (60 °C) and relative humidity (98%), up to 3000 h, on the properties of constituents of bio-based FRP composites. This research offers a distinctive contribution in terms of a comprehensive comparison of different fibre types: carbon, glass, basalt, and flax, subjected to the same simulated hygrothermal ageing. Additionally, the degradation of the sizing layer of each fibre type and its role in the loss of composite’s integrity was evaluated under identical manufacturing and testing conditions. A deeper knowledge about the fracture mechanisms of constituent materials is obtained by individually evaluating the mechanical and microstructural properties of the fibres, resin, and the fibre/matrix interface. The findings of this study will offer a comprehensive understanding of how different fibre types influence the degradation of bio-based polymer composites exposed to prolonged hygrothermal environments for their reliable and efficient application in structures exposed to aggressive environmental conditions.

## 2. Materials and Methods

### 2.1. Materials

**Figure 1:**
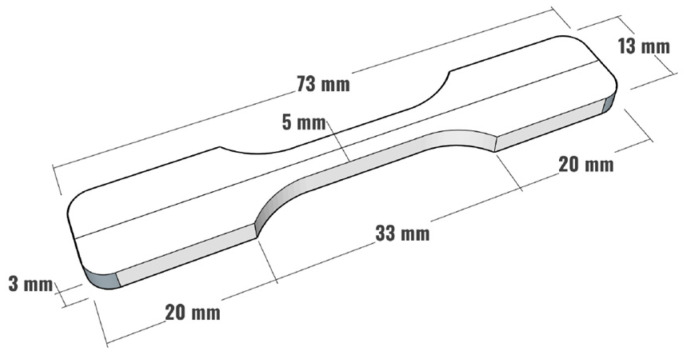
Dimensions of single yarn composite sample.

Four different types of fibre yarns (carbon, E-glass, basalt, and flax), shown in Figure 1, supplied by Colan Australia, were used to have four different variations in bio-epoxy composites. The physical properties of the fibre yarns supplied by the manufacturer are listed in Table 1. E-glass fibre yarns and basalt fibre yarns were coated with special sizing based on the universal silane coupling agents. Additionally, carbon fibre yarns were coated with an epoxy size, which provides better compatibility for use with epoxy resins. Furthermore, flax fibre yarns were made from 100% natural flax fibres without any additional sizing.

The bio-epoxy resin utilized for sample manufacturing was sourced from Change Climate Pty Ltd., Adelaide, SA, Australia, produced from a renewable, bio-based glycerol core, rather than a toxic Bisphenol A core sourced from crude oil. It is a non-toxic, aliphatic, two-part system having a mix ratio of 77:23 by weight. The physical, thermal, chemical, mechanical, and microstructural properties of bio-epoxy under hygrothermal exposure were evaluated previously in a separate study [35]. A summary of the critical parameters of the bio-epoxy resin after each exposure duration is listed in Table 2. Flexible silicone moulds were manufactured using liquid silicone (1:1) for efficient sample production.

### 2.2. Specimen Preparation

**Figure 2:**
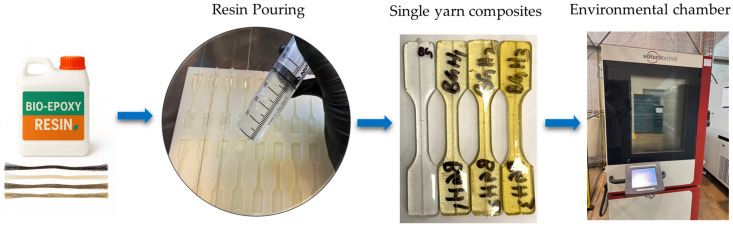
Sample manufacturing and hygrothermal exposure.

Dog-bone-shaped single yarn composite samples with the dimensions shown in Figure 1 were manufactured by combining bio-epoxy and four different fibre types. In the first step, the fibre yarns were aligned at the centre of silicone moulds with the help of guide notches, followed by sealing the notches with glue to avoid any sort of resin leakage, as shown in Figure 2. In the second step, a two-part bio-epoxy resin was mixed properly in the specified mixing ratios recommended by the manufacturer and was poured into silicone moulds. Finally, the prepared samples were fully cured by storing them at room temperature for 7 days, as recommended by the manufacturer. All the samples were prepared at the same time under similar conditions to maintain consistency and minimize variations.

### 2.3. Hygrothermal Conditioning

The single yarn composites and fibre yarns alone were placed in a simulated hygrothermal condition by using a hygrothermal chamber (C4-340, Weiss Technik, Balingen, Germany) to maintain a constant humidity (98%) and temperature (60 °C) for a period of 1000 h, 2000 h, and 3000 h, as shown in Figure 2. These environmental parameters were selected to simulate the ageing process, simultaneously replicating the realistic in-service thermal conditions that composites may experience throughout their service life [24].

### 2.4. Mechanical Characterization

**Figure 3:**
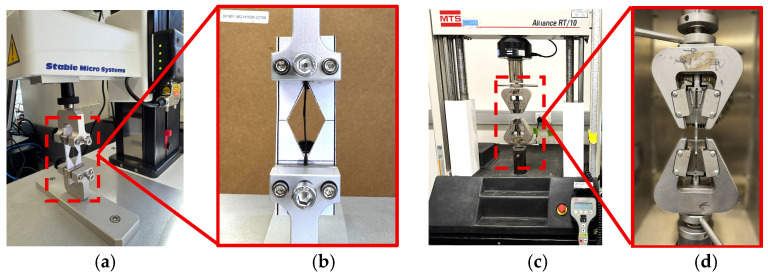
(**a**,**b**) Test setup for tensile test of fibre yarn using TA. XTplus100C texture analyzer (Stable Micro Systems, Godalming, UK), (**c**,**d**) Single yarn fragmentation test setup.

The tensile strength of control and aged fibre yarns (carbon, glass, basalt, and flax) was evaluated according to the ASTM C1557-20 [36]. The test was conducted in a fibre tensile tester with a load cell of 1kN and a test speed of 1 mm/min, as shown in Figure 3a,b. Single yarn fragmentation tests were performed using a tensile testing machine (Alliance RT/10, MTS systems corporation, Eden Prairie, MN, USA) with a 10 kN load cell under a loading rate of 1 mm/min, shown in Figure 3c,d. A minimum of five specimens for each sample type were tested after every ageing dilation to ensure statistical reliability of the results. A summary of the tests, including the corresponding standards and sample counts, is provided in Table 3.

### 2.5. Chemical Characterization and Microscopic Observations

The elemental composition at the surface of unaged and aged fibre yarns was obtained by employing energy dispersive X-ray (EDX) spectroscopy using the EDX mode of JEOL JCM-7000 Neoscope (JEOL Ltd., Tokyo, Japan). The fundamental focus of this analysis was to compare the oxygen-to-carbon ratios (O/C) of fibres before and after exposure to detect any chemical degradation of fibre sizing. The elemental composition of samples dried overnight at 120 °C was quantified at three different points. Microscopic analysis was performed using a JEOL JCM-7000 Neoscope to observe damage at the fibre surface or sizing layer, and at the fibre/matrix interface, after hygrothermal ageing.

## 3. Results and Discussion

### 3.1. Effect of Hygrothermal Conditioning on Tensile Behaviour of Fibres

#### 3.1.1. Tensile Strength

**Figure 4:**
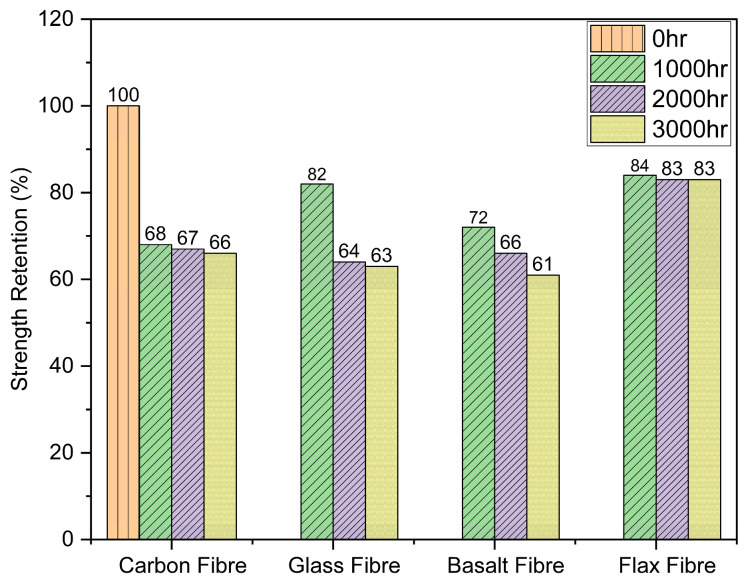
Tensile strength retention of fibre yarns after hygrothermal ageing.

The tensile strength of fibre yarns alone was adversely affected after prolonged exposure to a hygrothermal environment, as summarized in Table 4. A reduction of 34%, 37%, 39% and 20% in tensile strength was noticed in carbon, glass, basalt, and flax fibre yarns, respectively, after 3000 h of direct hydrothermal exposure. Figure 4 shows that in the case of carbon and flax yarns, the tensile strength dropped to 68% and 84%, respectively, after 1000 h, and only a slight decrement was noticed afterwards. While in the case of glass fibres, the strength dropped to 82% after 1000 h and further dropped to 64% at 2000 h without any further decrement with increased exposure time. The basalt fibres exhibited a progressive strength reduction with the passage of exposure time.

Despite the vulnerability of natural fibres to water absorption, the flax fibre yarns have shown better resistance to hygrothermal conditioning as compared to all the synthetic fibres. A possible reason for this is the presence of a sizing layer on the synthetic fibres, whereas flax yarns were composed of 100% natural fibres without any epoxy or silane-based chemical sizing; only a minimal textile finish may have been present from fibre processing. The sizing is normally applied for processability, enhancement of strength and durability during the weaving process, protecting them from breakage due to stretching, strain, and abrasion [34]. The sizing layer is the most critical component, and the deterioration of this sizing layer had an important contribution in the decline of tensile strength [37]. Carbon fibres were coated with epoxy sizing, which was damaged because of the hydrolysis of the epoxy sizing exposed to a hygrothermal environment, resulting in a 34% loss in strength. Both glass and basalt fibres have demonstrated the same extent of strength reduction after exposure because of the same silane-based sizing. The strength reduction in the glass fibre was attributed to the removal of the fibre sizing and the hydrolysis reaction of siloxane (Si-O-Si) bonds. Various researchers have reported that the degradation of the silane-based sizing layer occurs when exposed to elevated temperatures and hygrothermal conditions because of the hydrolysis of Si-O-Si bonding between fibre and sizing layer [31,37,38,39,40]. Zhong et al. [32] and Nikforooz et al. [31] reported a decrement of 29% and 42% in the tensile strength of E-glass fibres subjected to water and a temperature of 80 °C and 70 °C, respectively. They attributed this reduction to the damage of fibre sizing. In the case of basalt fibres, the dominant degradation mechanism was the leaching of oxides, mainly the reduction in (SiO_2_), as also reported by Gue et al. [41]. The considerable strength loss in synthetic fibres was attributed to the deterioration of the protective layer provided by fibre sizing. This reduction in strength would substantially decrease the tensile performance of bio-epoxy composites. In the case of FRP composites, the fibres are generally less affected by temperature and moisture, due to a protective resin layer that reduces the moisture ingression [42,43]. However, after prolonged exposure, there is a possibility that water will ingress and reach the fibres, leading to compromised mechanical performance of composites. Moisture ingress and elevated temperature can lead to plasticization, hydrolysis, or erosion of the sizing layer, thereby reducing interfacial bond and reducing load transfer efficiency. As a result, interfacial debonding occurs at the fibre/matrix interface due to the development of stress concentrations. Similar behaviour has been reported by different researchers [31,40,44], where damage occurred in the sizing layer under moisture and elevated temperature conditions resulted in reduced interfacial shear strength and tensile performance of composites.

#### 3.1.2. Fracture Behaviour

**Figure 5:**
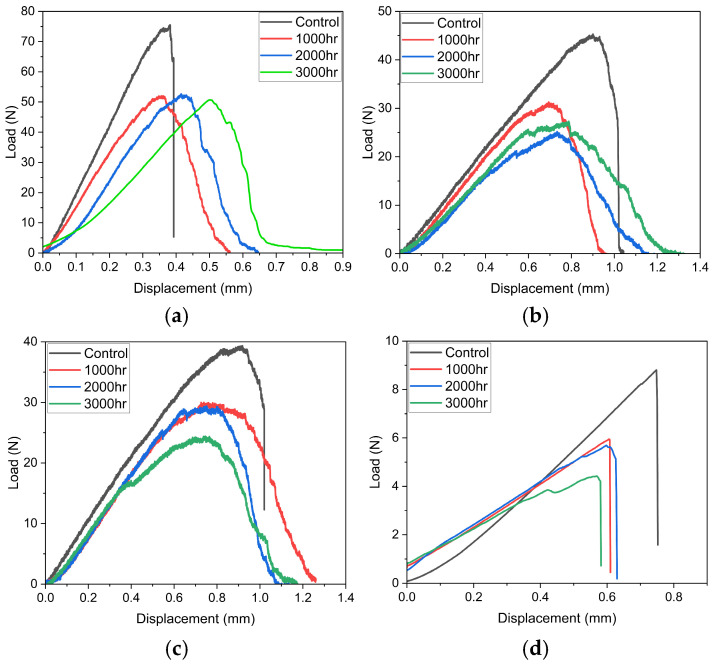
Failure Behaviour of (**a**) Carbon fibre yarn, (**b**) Glass fibre yarn, (**c**) Basalt fibre yarn, (**d**) Flax fibre yarn.

The load displacement curves of fibre yarns alone, shown in Figure 5, exhibited a prominent shift in failure behaviour after hygrothermal ageing. In all the synthetic fibres (Figure 5a–c), the control samples exhibited a linear rise followed by an abrupt drop, demonstrating a brittle failure. In fibre yarns, one of the roles of sizing is the adhesion of many constituent fibres, so that the yarn behaves as a single cohesive structure. This behaviour was observed because the sizing layer held the fibres together, and a bundle-dominated failure occurred, in which all the individual fibres shared the load efficiently up to their maximum capacity and fractured simultaneously. In contrast, the samples after conditioning (1000 h, 2000 h, and 3000 h) exhibited a reduced initial slope, a lower and broader failure peak, followed by an extended post-peak region. The hygrothermal exposure facilitated the removal/damage of the fibre sizing, causing the loss of adhesion between fibres and reduced inter-filament friction. As a result, the failure was governed by the fracture of individual filaments with less efficient load sharing and progressive damage as fibres break in a distributed manner without a global collapse. The severity of this progressive failure behaviour increased with increased exposure duration. Overall, the exposure transformed the yarn failure from a brittle bundle-dominated failure to a progressive filament-dominated failure.

**Figure 6:**
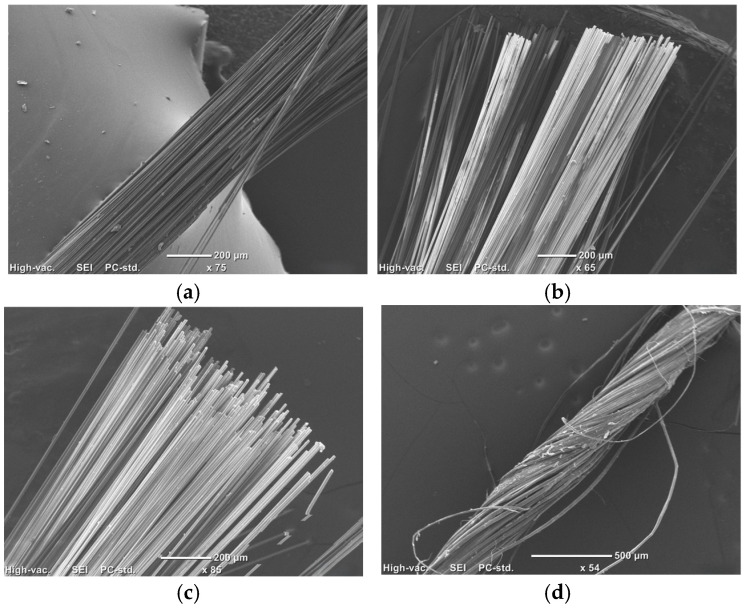
Comparison of fibre yarn structures: Untwisted yarns (**a**) Carbon fibre yarn, (**b**) Basalt fibre yarn, (**c**) Glass fibre yarn, and Twisted (**d**) Flax fibre yarn.

For flax fibres, the load displacement curves (Figure 5d) of unexposed yarns exhibited a sharp failure peak with an abrupt vertical drop representing a brittle bundle-dominated fracture. Unlike synthetic fibre yarns, which are all straight, flax yarns were twisted (Figure 6) and lacked a sizing layer, so the load sharing between fibres was governed by dry friction and mechanical interlocking due to the twists. Hygrothermal conditioning considerably reduced the peak load and displacements at failure, with a more pronounced effect at higher exposure duration (3000 h). Unlike synthetic fibres, the failure behaviour after exposure remained the same because the twisted yarn behaved as a single cohesive body even after exposure, with abrupt bundle-dominated failure with reduced strength.

#### 3.1.3. Fibre Damage Mechanisms

**Figure 7:**
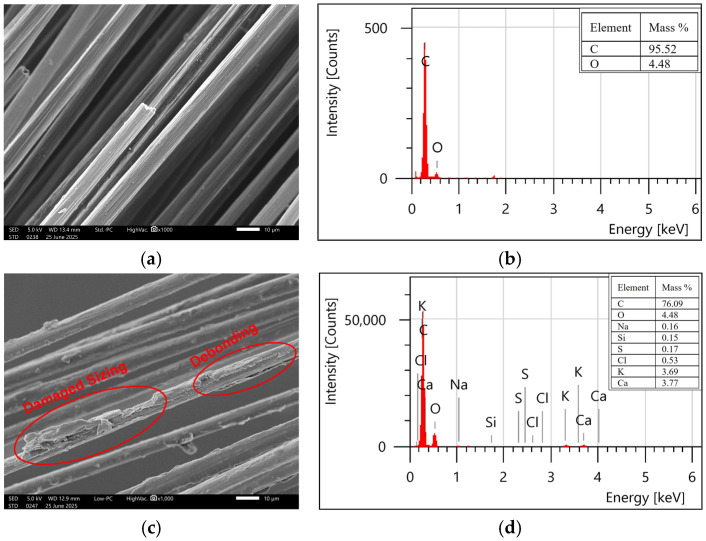
Surface morphology and elemental composition (SEM-EDS) of carbon fibres: (**a**,**b**) unexposed and (**c**,**d**) exposed.

SEM observations of the fibre surface were conducted before and after exposure for 3000 h to analyze the degradation of the fibres. EDX spectroscopy was also employed to identify the degradation mechanism governing the deterioration of the fibres. SEM images and EDS spectra of unaged and aged carbon fibres are shown in Figure 7. It can be noticed that carbon fibres have a smooth texture before exposure, but after exposure, significant erosion of epoxy sizing and debonding of the sizing layer from the fibre are visible. EDS spectra before exposure only detected carbon and oxygen (95.52% and 4.48%, respectively), confirming the rich epoxy layer, whereas after exposure, there were traces of some other elements as well. The oxygen to carbon (O/C) ratios were estimated using the mass percentages to reveal any oxidation and hydrolysis of the sizing layer, as presented in Table 5. An increase in the O/C ratio can be referred to as an indication of the oxidative degradation or hydrolysis of polymers. Jean et al. [45] employed a similar approach to identify the hydrolysis at the surface of carbon fibres under solar ultraviolet (UV) exposure. It was observed that O/C was increased from 0.04 to 0.20, indicating the occurrence of hydrolysis in the epoxy sizing layer.

**Figure 8:**
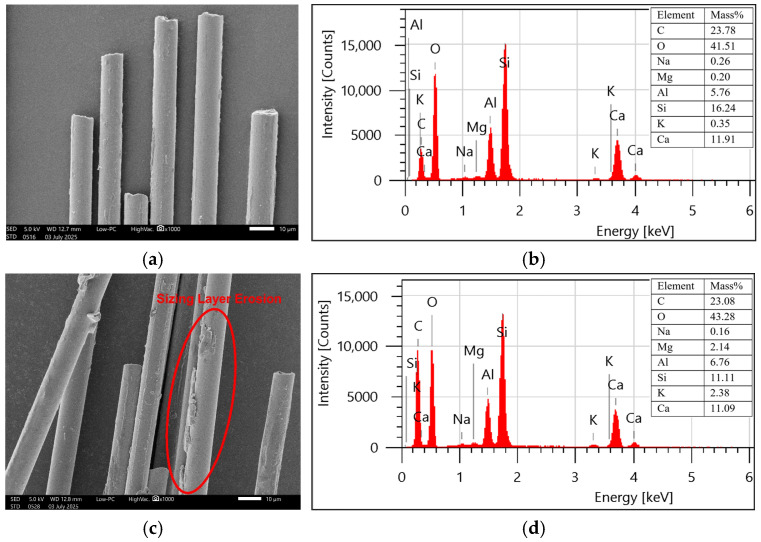
Surface morphology and elemental composition (SEM-EDS) (**a**,**b**) unexposed and (**c**,**d**) exposed glass fibres.

**Figure 9:**
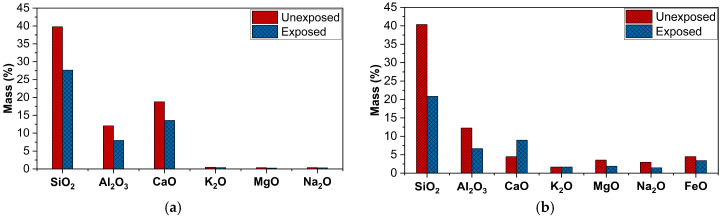
Oxide compositions of (**a**) Glass fibres (**b**) Basalt fibres.

**Figure 10:**
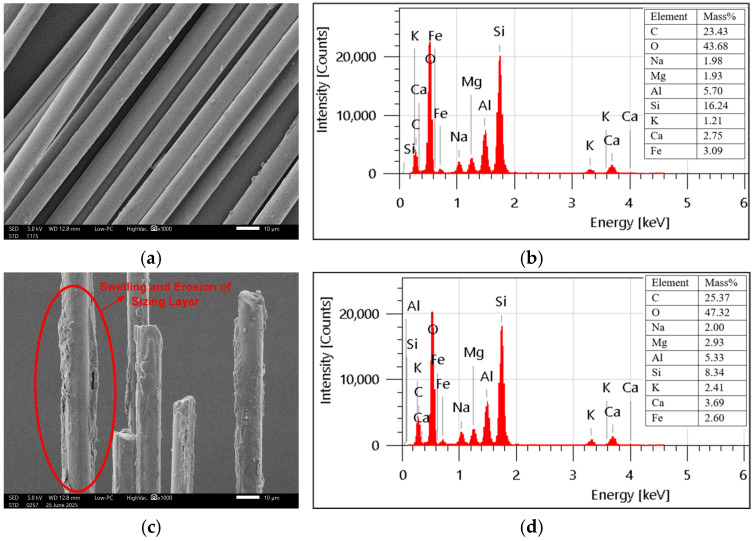
Surface morphology and elemental composition (SEM-EDS) of basalt fibres: (**a**,**b**) unexposed and (**c**,**d**) exposed.

The glass fibres had a smooth texture before exposure, but after exposure, erosion of the sizing layer can be observed because of the action of moisture and temperature, as evident from Figure 8. The carbon percentage was slightly reduced from 23.78% to 23.04%, while oxygen content was increased from 41.51% to 43.28%. Therefore, O/C ratio slightly increased from 1.74 to 1.87 after the exposure, indicating the occurrence of a hydrolysis reaction of Si-O-Si bonds [34]. The damage can also be attributed to the leaching and wash-off of silane-based sizing due to elevated temperature and moisture. It can be evidenced from the changes in oxide composition shown in Figure 9a that the amount of SiO_2_ has been significantly reduced from 39% to 27% after exposure, corresponding to the leaching of the sizing layer. A similar damage mechanism was observed in basalt fibres, as both glass and basalt have the same sizing. Figure 9b demonstrates the significant reduction of SiO_2_ percentage from 40% to 20%, resulting in the leaching-dominated degradation mechanism. SEM images (Figure 10) revealed the swelling and erosion of the sizing layer after exposure to a hygrothermal environment. EDS analysis showed that there was no hydrolysis detected as the O/C ratio remained unchanged after exposure, as shown in Figure 10. Gue et al. [41] also observed damage in basalt fibres due to the washing out of SiO_2_, as indicated by a reduced fraction after exposure to seawater at elevated temperatures.

**Figure 11:**
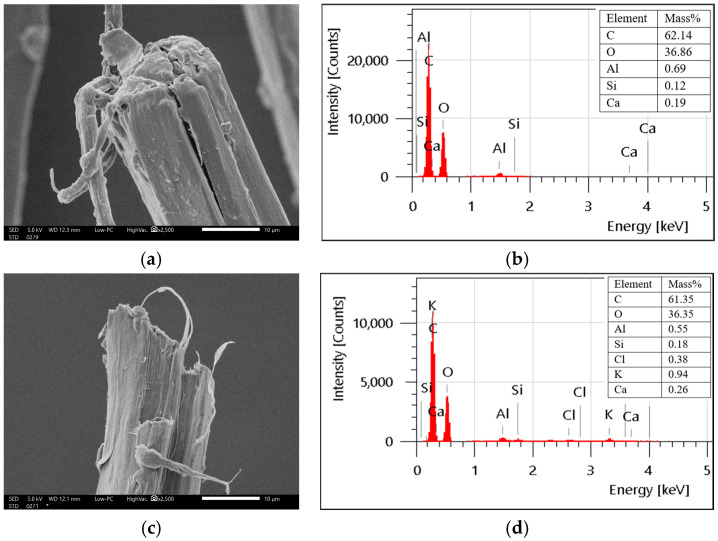
Surface morphology and elemental composition (SEM-EDS) of flax fibres (**a**,**b**) unexposed and (**c**,**d**) exposed.

Unlike synthetic fibres, flax fibres do not have any sizing layer. Therefore, the damage caused to these fibres was primarily due to water absorption, resulting in dimensional instabilities such as swelling and cracking, as revealed by the SEM micrographs in Figure 11. Swelling in flax fibres because of moisture absorption has been reported in previous studies [10,42,46]. Lu et al. [42] has quantified the swelling in the flax fibres at a relative humidity of 90% and observed swelling of 15% and 25% in elementary and technical flax fibres, respectively. There was no chemical damage as the O/C ratio remained the same before and after exposure (Table 5). Hence, the degradation was attributed to hygro-swelling and microcracking rather than chemical degradation, indicating potential for bio-composites with stable environmental performance.

In conclusion, the failure mechanism after hygrothermal ageing was fibre-specific and different for each fibre type as presented in Table 6. The deterioration of carbon fibres was primarily governed by hydrolytic/oxidative removal of the epoxy sizing. For glass fibres, the damage was dominated by Si–O–Si bond hydrolysis at the silane interface, combined with the washing out of silane sizing. Basalt fibres experienced damage because of the leaching-dominated loss of silica with minimal hydrolysis. Unlike synthetic fibres, in flax, the dominant damage mechanism was moisture-driven swelling and microcracking rather than chemical change. Collectively, these observations underline that synthetic fibres fail primarily through sizing deterioration, whereas natural fibres degrade through physical instability induced by water uptake. 

### 3.2. Effect of Hygrothermal Agaeing on IFSS

The interfacial shear strength was evaluated by applying the single yarn fragmentation approach, using the mathematical expression given below as Equation (1) [47]:

(1) $$\tau =\frac{F}{L\pi D}$$

where

*F* represents the load at failure, *L* is the length of the embedded fibre, and *D* is the fibre diameter. Table 7 presents the retention of IFSS after 3000 h of hygrothermal exposure and the average IFSS values after each exposure duration.

Hygrothermal conditioning has adversely influenced the interfacial shear strength across all the fibre types. Bio-epoxy composites reinforced with carbon fibres exhibited the lowest strength loss of only 4% after 3000 h. Bio-epoxy composites with glass and basalt exhibited similar decrements of 8% and 7%, in interfacial shear strength, respectively. Bio-epoxy/flax composites demonstrated the highest decrease in IFSS among all, with a reduction of 10% after 3000 h. In terms of absolute values, flax yarn has shown the highest IFSS values before and after exposure, but in relative comparison, carbon exhibited the highest IFSS retention.

**Figure 12:**
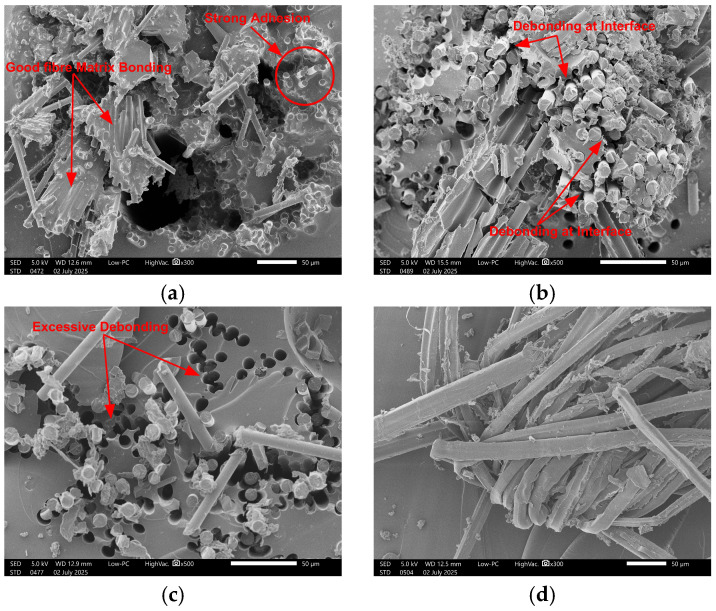
Morphology of fractured surface of bio-epoxy composites (**a**) carbon, (**b**) basalt, (**c**) glass, (**d**) flax.

The reduction in IFFS can be attributed to fibre/matrix damage caused by microcracking and debonding [43,48] because of the potential moisture ingression route [49]. In carbon/bio-epoxy composites, the reduction was mainly due to the hydrolysis of epoxy sizing present between the carbon fibre and matrix. Despite the higher vulnerability to damage because of hydrolysis, these composites exhibited a high IFSS retention of 96%. This behaviour can be attributed to good compatibility between epoxy sizing and bio-epoxy resin, resulting in stronger adhesion and less moisture ingress in the interfacial region. It can be observed from the SEM micrographs shown in Figure 12 that carbon fibres have very strong adhesion to resin, and the interface is intact even after exposure to hygrothermal conditions. At some points, whole fragments of resins are observed to be broken, but fibres were still firmly anchored in the resin. It can be concluded that the dominant failure mechanism was the plasticization of resin rather than the interface debonding.

For glass/bio-epoxy, the main cause of reduction in IFSS was debonding at the interface, facilitated by the hydrolysis of the sizing layer. The reduction in IFSS was also influenced by the hydrolysis of interfacial bonds linking the silane sizing and resin [31,40]. Wang et al. [44] also reported the damage of the sizing layer in glass fibre composites after hygrothermal ageing. Another reason was the poor compatibility of silane-based sizing with bio-epoxy, which has facilitated the excessive water ingress in the interfacial zone, causing the hydrolysis and leaching out of silica content, as evidenced by EDS for glass fibres alone in the previous Section 3.1.3. Basalt/bio-epoxy exhibited a strength retention of 93% after 3000 h, which is very close to that of glass/bio-epoxy (92%). This might be attributed to the fact that both the fibres share the same type of sizing layer. SEM micrographs (Figure 12b,c) revealed that the governing mechanism behind the degradation of glass/bio-epoxy and basalt/bio-epoxy was the debonding at the fibre/resin interface. Furthermore, it can also be observed that the extent of damage in glass/bio-epoxy was more prominent compared to basalt/bio-epoxy, which aligns with the mechanical results.

For flax/bio-epoxy, the absolute IFSS values were higher among other fibre types because of the strong polar interactions and mechanical interlocking with bio-based epoxy facilitated by the twisted yarn structure and the micro-fibrillated cellulose surface of flax fibres. Another possible reason might be the penetration of resin into the fibre lumen, resulting in reduced moisture ingress and better impregnation. Lu et al. [42] observed the penetration of polyester resin into the flax lumen, while Dhakal et al. [50] reported that the flax fibres have permeable walls allowing the resin intrusion. However, the bio-epoxy/flax composites exhibited the highest reduction in IFSS (10%) after hygrothermal ageing. Owing to the hydrophilic nature of flax, the cell wall allowed the moisture ingress through lumens and pits, causing the swelling and shrinkage cycles, resulting in the local stresses at the interphase. Lu et al. [42] reported the microcracking at the interface because of the swelling of 2.5–2.7% in flax composites after exposure to 90% relative humidity. Additionally, water replaces the hydrogen bonds between polymer chains and cellulose by making bonds with polymers and cellulose, eventually reducing the adhesion between fibre and resin. Thus, the fibre swelling and bond replacement by water might have increased the interfacial slip, causing the pull-out of yarn and finally fibre breakage. This governing failure mechanism of slippage and pull-out can also be examined in the SEM micrographs shown in Figure 12d.

In conclusion, hygrothermal ageing has triggered various fracture mechanisms dependent on the specific fibre type. The failure in carbon/bio-epoxy was governed by matrix plasticization, whereas the interface was largely preserved because of the better adhesion and bonding between epoxy sizing and resin. The glass/bio-epoxy failed primarily due to interfacial debonding caused by the hydrolysis of the silane coupling layer and silica depletion. Basalt/bio-epoxy showed the same debonding mechanism but with lower severity because no hydrolysis of the sizing layer was detected after exposure. Flax/bio-epoxy failed because of the fibre swelling and hydrogen-bond exchange at the interphase, which increased interfacial slip, yarn pull-out, and local fibre fracture. Overall, the degradation of bio-based epoxy composites reinforced with synthetic fibres was controlled by the interface, whereas in the case of flax, it was fibre-driven.

### 3.3. Statistical Evaluation of Influence of Fibre Types on Durability (ANOVA)

Statistical analysis was conducted using two-way Analysis of Variance (ANOVA) to verify whether fibre types have a significant effect on the durability of bio-epoxy composites or not. The independent variables considered in the ANOVA were fibre types and ageing time, whereas the dependent variables included the IFSS retention and retention of fibre yarn strength after exposure for 3000 h. The results presented in Table 8 clearly depicted that after hygrothermal ageing, the effect of fibre type and duration on the decrement of IFSS and tensile strength was significant, as the *p*-value for both is lower than 0.05 (i.e., 95% confidence level). The significant effect of fibre type can be referred to the variation in the fibre/resin interface because of the varying fibre surface nature and the types of sizing applied to each fibre type. Likewise, the significant influence of exposure time reflects the progressive nature of hygrothermal ageing, where prolonged exposure promotes moisture diffusion into the matrix along the interface, leading to plasticization of the resin and interfacial debonding.

It can also be concluded from the size effect (R^2^ values) that the changing fibre type is more damaging to the reduced durability of composites compared to the increase in the exposure time. This can be attributed mainly to the variations in the characteristics of the fibre/resin interface, fibre surface chemistry, and moisture affinity among fibre types. For instance, natural fibres absorb more moisture because of their hydrophilic nature, resulting in a weak fibre-matrix bond. In contrast, synthetic fibres are relatively less permeable with a sizing layer making a stronger bond with resin, resulting in greater interfacial stability. Therefore, while exposure duration mainly controls how long these degradation processes act, the fibre type fundamentally governs the extent and mechanism of degradation, making it the dominant factor influencing long-term durability.

The statistical analysis indicated that both fibre type and exposure duration significantly influenced the mechanical degradation of bio-epoxy composites conditioned to a hygrothermal environment. These findings align with Benmokrane et al. [51], who also reported a significant effect of fibre type, fibre sizing and resin chemistry on the ILSS and tensile strength of carbon, basalt, and glass FRP bars after simulated alkaline exposure at 60 °C. Sakuraba et al. [52] also demonstrated that fibre types have a substantial influence on the tensile strength and ILSS of FRP composites. Similarly to their findings, the present study demonstrated that the intrinsic properties of fibre yarns, such as surface chemistry, hydrophilicity, and sizing type, largely influenced the tensile strengths of fibre yarns interfacial degradation of composites.

The statistical results highlighted fibre type as a significant factor influencing mechanical performance under hygrothermal ageing. The durability trends observed in this study provide useful guidelines for material selection in structural applications expected to be exposed to moisture and elevated temperatures. Better interfacial stability of carbon/epoxy composites under hygrothermal conditions suggests their suitability for durability-critical components in infrastructure applications. Glass and basalt fibre composites exhibiting moderate performance may remain a suitable option for applications where cost efficiency is prioritized along with an adequate level of performance. In contrast, the moisture sensitivity and higher interfacial damage observed in flax fibre composites highlight the need for surface modification, or environmental protection strategies if natural fibres are to be adopted in structural applications.

## 4. Conclusions

This research evaluated the mechanical, chemical, and microstructural characteristics of single yarn bio-epoxy composites with carbon, glass, basalt, or flax yarn subjected to hygrothermal ageing. The composites were conditioned at 98% RH and 60 °C for 1000, 2000, and 3000 h. The present study offers a comprehensive comparison of the response of different fibre types towards a prolonged hygrothermal environment. Although fibre yarns alone showed massive strength reduction after direct exposure, bio-epoxy composites showed excellent performance under hygrothermal exposure. The findings demonstrated the environmental durability of bio-epoxy composites, showing that sustainable matrix systems can maintain integrity. From the key findings, the following conclusions were established:

1. The tensile strength of fibre yarns alone was adversely affected after hygrothermal exposure. The reduction was more prominent in synthetic fibres (34% in carbon; 37% in glass; 39% in basalt) because of the chemical degradation of the sizing layer, as evidenced by SEM and EDS. Flax retained higher tensile strength (83%) than synthetic fibres, as there was no chemical degradation and damage occurred only due to swelling and micro-cracks.
2. The failure behaviour of fibre yarns was altered after hygrothermal exposure. The loss of adhesion between fibres within the yarn because of the damage of sizing in the synthetic fibres resulted in a progressive failure at reduced peak loads after exposure, unlike an abrupt failure at higher loads before exposure. The failure behaviour was not affected for flax fibres, which was a bundle-dominated failure facilitated by the swelling and cracking of fibres after moisture absorption.
3. Despite ageing, interfacial shear strength was largely preserved in bio-epoxy composites, with the highest retention of up to 96% for carbon because of the better compatibility of epoxy sizing with resin, resulting in stronger adhesion and better interfacial bond. Glass and basalt exhibited a retention of 92% and 93%, respectively, whereas flax composites showed the lowest retention of 90%, attributed to moisture-induced swelling and weaker fibre–matrix adhesion.
4. The matrix failure was the dominant fracture mechanism in bio-epoxy composites with carbon, while fibre/resin debonding was dominant for glass and basalt composites. This can be due to the better adhesion between carbon and bio-epoxy, but in glass and basalt composites, the excessive debonding was caused by the hydrolysis of the interface. In the case of flax, the fracture was dominated by fibre breakage and pull-out. These fracture mechanisms were visualized and confirmed through SEM.
5. SEM and EDS analyses confirmed that hygrothermal exposure caused fibre-specific damage mechanisms resulting in reductions in tensile and interfacial shear strength. In synthetic fibres, the hydrolysis and leaching of the sizing layers were mainly responsible for degradation. Flax fibres exhibited only physical swelling and microcracking, with no evidence of chemical change.
6. Two-way ANOVA confirmed significant effects of both fibre type and exposure duration on property retention, with larger effect sizes for fibre type. Tensile properties of fibre yarns were more sensitive than IFSS because in composites, fibres were protected by resin.

The conclusions drawn from this work are specific to the selected fibre and resin combinations, as well as the parameters evaluated for each property. Nevertheless, the methodology and outcomes presented here provide an outline for assessing the durability and degradation of bio-composites incorporating different fibre types subjected to different environmental conditions. Future studies could apply this methodology to alternative fibres and resins to authenticate the reliability of the existing prediction models and to advance the understanding of the durability behaviour of polymer composites under hygrothermal exposure.

## Figures and Tables

**Table 1:** Physical properties of yarns provided by the supplier.

Physical Properties	Carbon Fibre Yarn	Glass Fibre Yarn	Basalt Fibre Yarn	Flax Fibre Yarn
Density [g/cm^3^]	1.78	2.54	2.70	1.54
Tex [gm/km]	68	68	70	36
Diameter [µm]	216.9	189.7	179.9	147.1

**Table 2:** Summary of properties of bio-epoxy resin subjected to hygrothermal ageing [35].

Properties	Exposure Duration				% Retention After 3000 h
	0 h	1000 h	2000 h	3000 h	
Water Absorption (%)	0%	8.4%	9.3%	9.5%	---
Degree of cure (%)	99.9%	99.9%	99.9%	99.9%	---
Glass transition temperature (°C)	44.4 °C	44.2 °C	47.5 °C	46.6 °C	105%
Tensile strength (MPa)	52.3	50.1	49.6	47.2	90.2%
Modulus (MPa)	2972	2688	2589	2569	86.4%
Strain at failure (%)	3.84	4.24	3.95	4.46	116.1%

**Table 3:** Relevant standards and number of samples.

Properties	Number of Samples			
	0 h	1000 h	2000 h	3000 h
Tensile Strength of Fibre Yarns (ASTM C1557-20 [36])				
Carbon fibre	5	5	5	5
Glass fibre	5	5	5	5
Basalt fibre	5	5	5	5
Flax fibre	5	5	5	5
Interfacial Shear Strength (IFSS)				
Bio-epoxy carbon composites	5	5	5	5
Bio-epoxy glass composites	5	5	5	5
Bio-epoxy basalt composites	5	5	5	5
Bio-epoxy flax composites	5	5	5	5
Chemical Analysis (EDS)				
Fibre yarns (each)	3	3	3	3
Microstructural Properties (SEM)				
Fibre yarns (each)	3	3	3	3
Single yarn composites (each)	3	3	3	3

**Table 4:** Reduction in tensile strength after hygrothermal ageing.

Fibre Type	Parameter	Exposure Time				Retention After 3000 h
		0 h	1000 h	2000 h	3000 h	
Carbon	Tensile Strength (MPa)	1971	1337	1326	1308	66%
	CoV (%)	3.50	4.98	4.11	3.38	
Glass	Tensile Strength (MPa)	1602	1314	1032	1004	63%
	CoV (%)	2.97	3.39	4.45	3.01	
Basalt	Tensile Strength (MPa)	1518	1091	1003	928	61%
	CoV (%)	4.42	3.69	4.93	2.76	
Flax	Tensile Strength (MPa)	741	626	613	596	80%
	CoV (%)	4.88	3.27	4.43	4.25	

**Table 5:** Comparison of oxygen to carbon (O/C) ratios for unexposed and exposed fibres, illustrating changes in surface composition.

Fibre Type	Unexposed			Exposed		
	C%	O%	O/C	C%	O%	O/C
Carbon	95.52	4.48	0.04	76.09	15.44	0.20
Glass	23.78	41.51	1.74	23.08	43.28	1.87
Basalt	23.43	43.68	1.86	25.37	47.32	1.86
Flax	62.14	36.86	0.59	61.35	36.35	0.59

**Table 6:** Dominant failure mechanisms in different fibre types.

Fibre Type	Dominant Failure Mechanism
Carbon Fibres	Hydrolytic/oxidative removal of epoxy sizing.
Glass Fibres	Si–O–Si bond hydrolysis at the silane interface, washing out of silane sizing.
Basalt Fibres	Leaching-dominated loss of silica with minimal hydrolysis.
Flax Fibres	Moisture-driven swelling and microcracking.

**Table 7:** Reduction in the IFSS of composite samples.

Fibre Type	Parameter	Exposure Time				Retention After 3000 h
		0 h	1000 h	2000 h	3000 h	
Carbon	IFSS (MPa)	36.89	36.47	35.97	35.25	96%
	CoV (%)	4.00	2.43	3.36	1.93	
Glass	IFSS (MPa)	45.08	43.26	42.50	41.88	92%
	CoV (%)	1.22	4.21	2.77	2.91	
Basalt	IFSS (MPa)	42.14	42.04	39.62	39.50	93%
	CoV (%)	4.00	3.23	4.35	4.11	
Flax	IFSS (MPa)	53.29	50.11	48.90	47.98	90%
	CoV (%)	3.00	1.53	3.02	3.51	

**Table 8:** Outcome of Two-way ANOVA.

Dependent Variable	Independent Variable	*p*-Value	R^2^
Retention of tensile strength	Fibre Type	<0.001	0.69
	Ageing Time	<0.001	0.33
Retention of IFSS	Fibre Type	<0.001	0.66
	Ageing Time	<0.003	0.22

## Data Availability

The original contributions presented in this study are included in the article. Further inquiries can be directed to the corresponding author.
